# Active Surveillance of Carbapenemase-Producing Organisms (CPO) Colonization With Xpert Carba-R Assay Plus Positive Patient Isolation Proves to Be Effective in CPO Containment

**DOI:** 10.3389/fcimb.2019.00162

**Published:** 2019-05-14

**Authors:** Menglan Zhou, Timothy Kudinha, Bin Du, Jinmin Peng, Xiaojun Ma, Yang Yang, Ge Zhang, Jingjia Zhang, Qiwen Yang, Ying-Chun Xu

**Affiliations:** ^1^Department of Clinical Laboratory, Peking Union Medical College Hospital, Peking Union Medical College, Chinese Academy of Medical Sciences, Beijing, China; ^2^Graduate School, Peking Union Medical College, Chinese Academy of Medical Sciences, Beijing, China; ^3^Beijing Key Laboratory for Mechanisms Research and Precision Diagnosis of Invasive Fungal Diseases, Beijing, China; ^4^Department of Clinical Laboratory, Charles Sturt University, Orange, NSW, Australia; ^5^Department of Medical Intensive Care Unit, Peking Union Medical College Hospital, Chinese Academy of Medical Sciences, Beijing, China; ^6^Department of Infectious Diseases, Peking Union Medical College Hospital, Chinese Academy of Medical Sciences, Beijing, China

**Keywords:** Xpert Carba-R assay, colonization, carbapenemase-producing organisms, infection control, screening

## Abstract

**Background:** Rapid screening of patients for colonization with carbapenemase-producing organisms (CPO), coupled with implementation of infection prevention strategies, has the potential to contain the spread of CPO.

**Methods:** We first evaluated the performance of Xpert Carba-R assay (in comparison with other phenotypic methods) for carbapenemase detection using clinical isolates, and then used it to determine the intestinal CPO colonization in hospitalized patients. We then assessed the effectiveness of patient isolation in controlling the spread of CPO in a medical intensive care unit.

**Results:** The Xpert Carba-R assay required the least processing time to reveal results and showed a 94.5% sensitivity and specificity in carbapenemase detection, except for IMP-8 (*n* = 4). During a 6-month study period, 134 patients in one ward were studied for CPO colonization and infection. Fifteen patients (11.2%) were colonized by CPO as detected by Xpert Carba-R assay, including three NDM, three IMP, and nine KPC possessing strains. The overall colonization and CPO infection rates were both 11.2% each. Isolation of patients with CPO led to a reduction in both colonization (from 28.6 to 5.6%) and infection rates (from 35.7 to 2.8%) during the study period (*p* < 0.05).

**Conclusion:** Active surveillance of CPO utilizing the Xpert Carba-R assay supplemented with immediate patient isolation, proved to be an effective strategy to limit the spread of CPO in a health care setting.

## Introduction

The rapid dissemination of carbapenemase-resistant organisms (CROs) has been reported worldwide, and is predominantly attributed to the production of carbapenemases (Tzouvelekis et al., 2012). Nosocomial transmission of carbapenemase-producing organisms (CPO) is a serious public health concern as invasive infections by these organisms are associated with a very high mortality rate (Lasserre et al., 2015; Rotova et al., 2017). Treatment options for CPO are limited as the organisms often exhibit widespread resistance to multiple antibiotics (Pannala et al., 2018). Colonization by CPO may promote infection, and carriers, particularly asymptomatic ones, may act as an important reservoir for CPO dissemination within the hospital setting. Furthermore, carriers of CPO are at higher risk of acquiring a subsequent infection (Borer et al., 2012). Rapid and accurate screening for CPO colonization among hospitalized patients, coupled with implementation of infection prevention strategies, including strict isolation practices, has the potential to interrupt the spread of CPO in hospitals (Wiener-Well et al., 2010; Singh et al., 2012).

Nevertheless, such interventions are critically dependent on accurate and rapid identification of patients colonized by CPO through laboratory testing. In this regard, the introduction of rapid and sensitive methodologies for the detection of CPO is of utmost importance. Unfortunately, detection of CPO still remains a challenge for most clinical microbiology laboratories in China. Currently, several phenotypic methods are available for CPO screening, including growth-based assays (modified Hodge test [MHT]) (Akhi et al., 2017), rapid colorimetric-based assays (manual and commercial versions of the Carba NP test) (Gauthier et al., 2017), matrix-assisted laser desorption/ionization time-of-flight mass spectrometry (MALDI-TOF MS)-based meropenem hydrolysis assays (MHA) (Papagiannitsis et al., 2015), immune-chromatographic lateral flow assays (Wareham and Abdul Momin, 2017), and most recently, the modified carbapenem inactivation method (mCIM) (Tamma et al., 2017). All of these assays are culture-based and thus limited to detection of bacterial strains.

The Food and Drug Administration (FDA)-cleared Xpert Carba-R (Cepheid, Sunnydale, CA) assay, on the other hand, is a multiplex PCR that detects the genes encoding for the 5 most common carbapenemases, including KPC, NDM, VIM, IMP, and OXA-48-like enzymes, both directly from bacterial colonies and from rectal swabs, in 2 easy steps that can be completed within 1 h (Moore et al., 2017). Several studies have been conducted in the United States and Europe to assess the accuracy of the Xpert Carba-R assay compared to conventional culture and DNA sequencing, in the detection of CPOs, with reported sensitivity and specificity ranging from 96.6 −100% to 94.2–100%, respectively (Genc et al., 2016; Oviano et al., 2016; Tato et al., 2016; Hoyos-Mallecot et al., 2017). To date, only one study has assessed the performance of Xpert Carba-R assay in determining the intestinal CPO colonization rates, and this was performed in an unselected intensive care unit cohort of patients in Korea. In that study, the assay exhibited good sensitivity, sufficient enough to detect CPO even when culture was negative (Kim et al., 2016). However, none of the previous studies have assessed the utility of combining CPO colonization detection (by Xpert Carba-R assay) with implementation of intervention measures, for CPO infection control.

Thus, in the present study, we conducted a two-stage study to assess the utility of the Xpert Carba-R assay in controlling the transmission of CPO in China. The first stage evaluated the performance of Xpert Carba-R assay (compared with other phenotypic methods) for carbapenemase detection using clinically obtained carbapenem resistant Enterobacteriaceae (CRE) isolates. In the second stage, we conducted an active CPO surveillance study by determining the intestinal CPO colonization rates among hospitalized patients using Xpert Carba-R assay (by directly testing rectal swabs of hospitalized patients), and coupled this with patient isolation in a medical intensive care unit (MICU) ward, in order to contain the spread of CPO.

## Materials and Methods

### Comparison of Xpert Carba-R Assay With Phenotypic Methods for CPO Detection

#### Bacterial Isolates

A total of 100 clinical non-duplicate CRE isolates from blood (*n* = 24), sputum (*n* = 21), midstream urine (*n* = 20), ascitic fluid (*n* = 6), abscess (*n* = 6), drainage fluid (*n* = 5), tissue (*n* = 5), wound (*n* = 3), bile (*n* = 2), catheter (*n* = 2), and various sterile and non-sterile sites (*n* = 6), referred by 34 teaching hospitals in China, to Peking Union Medical College Hospital (PUMCH), from 2004 to 2014, were included. All the *Enterobacteriaceae* isolates were identified by MALDI-TOF MS (Bruker Biotyper Bruker Daltonik, Bremen, Germany). CRE was defined as resistance to one of the carbapenems, imipenem (IPM), meropenem (MEM) or ertapenem (ETP), by broth microdilution method (BMD) according to the latest CLSI 2018 breakpoints criteria (Clinical Laboratory Standards Institute, 2018).

#### Molecular Detection of Carbapenemase Genes

*bla*_IMP_, *bla*_VIM_, *bla*_NDM_, *bla*_AIM_, *bla*_SPM_, *bla*_KPC_, *bla*_DIM_, *bla*_BIC_, *bla*_GIM_, *bla*_SIM_, and *bla*_OXA−48_ genes were detected by multiplex PCR as previously described (Poirel et al., 2011). *bla*_GES_, *bla*_NMC_, *bla*_SME_, and *bla*_IMI_ genes were each detected by a single primer set (Queenan and Bush, 2007). All positive isolates were sent for subsequent DNA sequencing. The obtained gene sequences were compared with those in the database located at NCBI blast server (http://blast.ncbi.nlm.nih.gov). A minimum of 99% sequence identity and 100% coverage threshold was deemed sufficient enough for confirmation of each gene (Pierce et al., 2017).

#### Method Comparison Study

All the CRE isolates were subcultured from frozen stocks onto 5% sheep blood agar (BAP) plate at 35°C for an overnight incubation, followed by a second subculture prior to phenotypic testing including MHT, Carba NP, mCIM and MHA, as previously described (Zhou et al., 2018). As for the Xpert Carba-R assay (Cepheid, Sunnyvale, USA), which detects the *bla*_KPC_, *bla*_NDM_, *bla*_VIM_, *bla*_OXA−48_, and *bla*_IMP−1_ carbapenem resistance genes in a GeneXpert cartridge, isolates to be tested were inoculated into the sample reagent and loaded into the cartridge following the manufacturer's instructions. The assay has a run time of 47 min in the instrument. Quality control was performed according to the Xpert package insert. Results were analyzed using GeneXpert Software Version 4.3 (AlTamimi et al., 2017; Miller et al., 2017). All the results generated by phenotypic tests and Xpert Carba-R assay were compared to reference DNA sequencing results.

#### Statistical Analysis

The investigators performing phenotypic testing were blinded to the identity of the isolates. Agreement and validity values were calculated with a 95% confidence interval (CI) based on an exact binomial distribution. Data were analyzed using trend Chi-square tests, SPSS, version 15.0 (SPSS Inc, Chicago, USA).

### Clinical Evaluation for Patient CPO Colonization Screening

#### Study Design

We performed a single-center prospective study from January 1st to June 30th 2017 in a Medical ICU (MICU) ward in PUMCH. The study was approved by the Human Research Ethics Committee of PUMCH and written informed consent was obtained from each patient or their relatives.

On the very first day (January 1) of the study, rectal swabs were collected from all the patients in the MICU ward and screened for CPO by Xpert Carba-R assay. After that, follow-up tests were performed for each patient upon admission, and at weekly intervals until discharge from MICU. Patients with a positive result were isolated in a designated room immediately. CPO colonization rates were calculated monthly based on Xpert Carba-R assay results. For each patient admitted in the ward at the time of the study, medical records were checked to determine if any CPO infection occurred during hospitalization. Xpert Carba-R assay was used for carbapenemase detection if any CRO was isolated from sterile sites.

#### CPO Screening Among Clinical Patients

A double swab set (Venturi Transystem; Copan, CA) and transport medium (liquid Stuart transport swab; Copan) were used to collect and transport rectal swab specimens from eligible subjects. One swab was inoculated onto a BAP and a China Blue Agar (CBA) plate (screen for gram-negative bacteria), and streaked for isolation followed by an overnight incubation at 35°C. Colonies growing on the CBA plate were identified by MALDI-TOF MS. Susceptibility testing to confirm carbapenem resistance was performed by standard disk diffusion method with ETP, IPM, and MEM, following the CLSI guidelines (Clinical Laboratory Standards Institute, 2018). If the presence of a carbapenem-non-susceptible organism was confirmed (intermediate or resistant) to ETP, IPM or MEM using the CLSI interpretive criteria in document M100-S28 (Clinical Laboratory Standards Institute, 2018), DNA was extracted and then purified, amplified and sequenced using primers as previously described (Moore et al., 2017). The other swab was used for the Xpert Carba-R assay as per the manufacturer's instructions. To minimize the potential bias of sampling differences, the two swabs were gently rolled against one another before starting the Cepheid Xpert Carba-R assay and culture procedure (Tato et al., 2016).

## Results

### Accuracy and Rapidity of Xpert Carba-R Assay in Comparison With Other Phenotypic Tests

Ten bacterial species were identified among 100 CRE, including 38 *Klebsiella pneumoniae*, 22 *Enterobacter cloacae*, 20 *Escherichia coli*, seven *K. oxytoca*, four *Citrobacter freundii*, three *Enterobacter aerogenes*, two each of *Serratia marcescens* and *K. planticola*, one each of *Proteus mirabilis* and *Providencia rettgeri*. A total of 73 (73%) isolates were positive for carbapenemase genes, among which 30 *Klebsiella pneumoniae* carbapenemase (KPC), 20 New Delhi metallo-β-lactamase (NDM), 19 Imipenem metallo-β-lactamase (IMP), two each of IMP & Verona integron-encoded metallo-β-lactamase (VIM) and KPC & IMP carbapenemase genes, were identified (Supplementary Table S1).

The results showed a notable variation between phenotypic assays and molecular-based Xpert Carba-R assay in terms of rapidity, sensitivity, specificity, positive predictive value (PPV), and negative predictive value (NPV), in detecting carbapenemase production among all CRE (Table 1). In the phenotypic assays, Carba NP, and MHA assays take 2–3 h to get results whilst MHT and mCIM take 16–24 h. The molecular-based Xpert Carba-R assay takes less than an hour to complete. The overall sensitivity and specificity for Xpert Carba-R assay was 94.5 and 100%, respectively. Using this method, a 100% sensitivity and specificity was observed for all the carbapenemase genes except IMP (sensitivity: 79.0%), for which four isolates possessing IMP-8 were not detected. However, this gene is not within the detection range of the Xpert Carba-R assay. The 2 h-incubation MHA exhibited the highest overall sensitivity, specificity, PPV and NPV of 100% each, followed by Carba NP and mCIM-MEM with a sensitivity of 98.6% each. In contrast, the 1 h-incubation MHA performed relatively poorly, with an overall sensitivity of 91.8%, mainly due to failure to detect KPC (Supplementary Table S1). Among three carbapenem disks using mCIM, MEM performed the best with the highest sensitivity (98.6%) and specificity (100%) compared to IPM (sensitivity: 97.3%; specificity: 96.3%) and ETP (sensitivity: 95.9%; specificity: 100%). The MHT assay performed the worst in CPO detection, with the lowest sensitivity (87.7%) and specificity (85.2%).

**Table 1:** Comparison of the performance of Xpert Carba-R assay vs. other phenotypic methods for carbapenemase detection using prospectively collected CRE isolates.

**Detection Methods**	**Sensitivity (95% CI)**	**Specificity (95% CI)**	**PPV (95% CI)**	**NPV (95% CI)**	**Time**
Xpert	94.5 (86.6–98.5)	100 (87.2–100)	100 (94.8–100)	87.1 (70.2–96.4)	47 min
IMP (*n =* 19)	79.0 (54.4–94.0)	100 (95.6–100)	100 (78.2–100)	95.3 (88.4–98.7)	
IMP+VIM (*n =* 2)	100 (15.8–100)	100 (96.3–100)	100 (15.8–100)	100 (96.3–100)	
KPC (*n =* 30)	100 (88.4–100)	100 (94.9–100)	100 (88.4–100)	100 (94.9–100)	
KPC+IMP (*n =* 2)	100 (15.8–100)	100 (96.3–100)	100 (15.8–100)	100 (96.3–100)	
NDM (*n =* 20)	100 (83.2–100)	100 (95.5–100)	100 (83.2–100)	100 (95.5–100)	
MHA−1 h	91.8 (83.0–96.9)	100 (87.2–100)	100 (94.6–100)	81.8 (64.5–93.0)	2 h
MHA−2 h	100 (95.1–100)	100 (87.2–100)	100 (95.1–100)	100 (87.2–100)	3 h
Carba NP	98.6 (92.6–100)	100 (87.2–100)	100 (95.0–100)	96.4 (81.7–99.9)	2–3 h
MHT	87.7 (77.9–94.2)	85.2 (66.3–95.8)	94.1 (85.6–98.4)	71.9 (53.3–86.3)	18–24 h
mCIM–MEM	98.6 (92.6–100)	100 (87.2–100)	100 (95.0–100)	96.4 (81.7–99.9)	18–24 h
mCIM–IPM	97.3 (90.5–99.7)	96.3 (81.0–99.9)	98.6 (92.5–100)	92.9 (76.5–99.1)	18–24 h
mCIM–ETP	95.9 (88.5–99.1)	100 (87.2–100)	100 (94.9–100)	90.0 (73.5–97.9)	18–24 h

*PPV, positive predictive value; NPV, negative predictive value; MHT, modified Hodge test; MHA, meropenem hydrolysis assay; mCIM, modified carbapenem inactivation method; MEM, meropenem; IMP, imipenem; ETP, ertapenem*.

### Xpert Carba-R Assay Results With Prospective Rectal Swab Specimens

A total of 134 patients were included in the study, and 350 rectal swabs were collected and screened for CPO with the Xpert Carba-R assay during the 6-month study period. Eighty-three (61.9%) of the patients were screened for CPO colonization at least twice during hospitalization (Supplementary Table S2). Of the 134 patients tested, 15 patients (corresponding to 51 specimens) were CPO positive by Xpert Carba-R assay, including three NDM, three IMP, and nine KPC. Conventional culture and DNA sequencing confirmed one NDM-producing and nine KPC-producing *K. pneumoniae* strains. For three cases involving IMPs, one of NDM, and one of KPC, no organisms were detected from swab culture. In another NDM positive rectal swab, no carbapenemase gene was detected from the carbapenem-sensitive but ESBL-positive *E. coli*.

Monthly CPO rectal colonization rates and infection rates in the MICU ward are shown in Tables 2, 3. The overall rectal colonization and CPO infection rates were both 11.2% each. At the beginning of the study, four out of 14 patients in the ward were CPO positive by Xpert Carba-R assay, contributing to a colonization rate of 28.6%. The four patients were isolated immediately. All the patients, including those newly admitted, were continually monitored for CPO colonization at weekly intervals, and all positive patients were isolated. Subsequent surveillance showed a significant decreasing trend in the colonization rate from 21.7% in January to 5.6% in June (*p* = 0.011) (Table 2).

**Table 2:** Monthly CPO rectal colonization rates based on Xpert Carba-R assay during the 6-month study period.

**Month**	**No. of Patients**	**Xpert Carba-R assay for rectal swabs**		**Rectal colonization rate (%)**	***p***
		**Pos**	**Neg**		
Baseline	14	4	10	28.6	0.011
January	23	5	18	21.7	
February	20	2	18	10.0	
March	23	3	20	13.0	
April	44	5	39	11.4	
May	39	3	36	7.7	
June	36	2	34	5.6	
Total	134	15	119	11.2	

*Pos: positive, Neg: negative*.

**Table 3:** Monthly CPO infection rates during the 6-month study period.

**Month**	**No. of Patients**	**No. of patients with CPO Infection**	**Xpert Carba-R assay for rectal swabs in CPO infected patients**		**CPO Infection Rate (%)**	***p***
			**Pos**	**Neg**		
Baseline	14	5	4	1	35.7	0.000
January	23	6	5	1	26.1	
February	20	3	2	1	15.0	
March	23	4	3	1	17.4	
April	44	5	5	0	11.4	
May	39	2	2	0	5.1	
June	36	1	1	0	2.8	
Total	134	15	13	2	11.9	

*Pos: positive, Neg: negative*.

**Figure 1:**
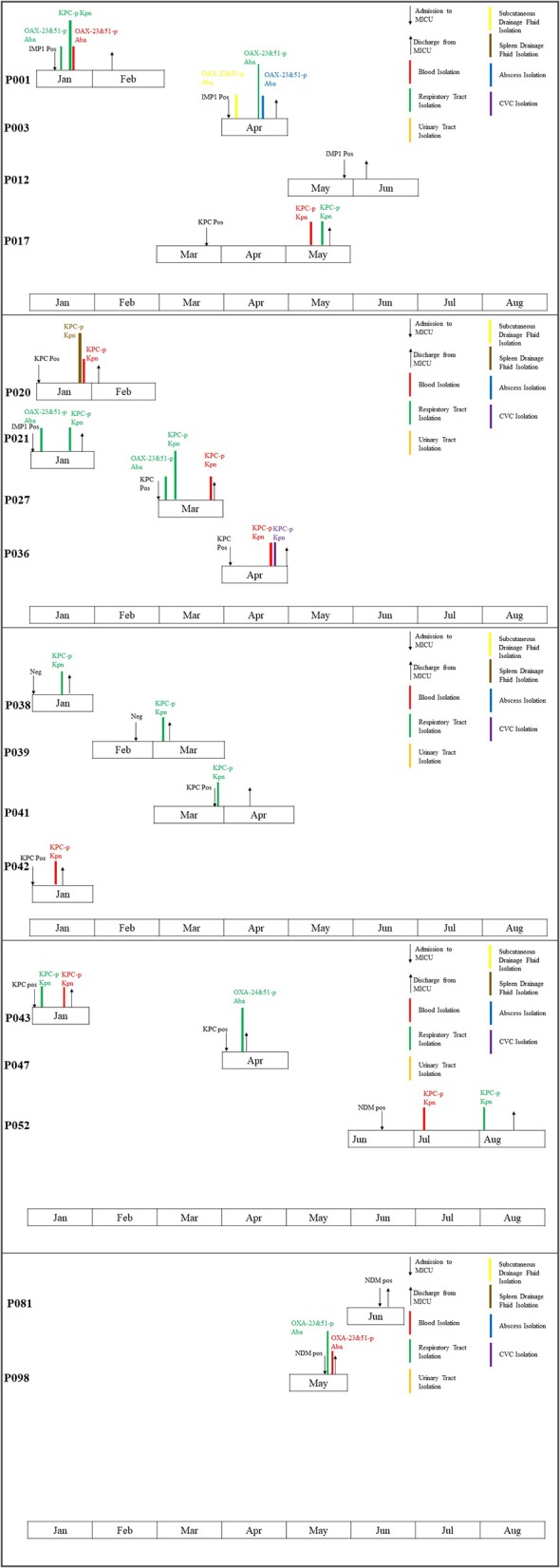
Timeline of events amongst 17 patients colonized and/or infected with CRO.

In 13 out of the 15 patients who tested positive for CPO colonization by Xpert Carba-R assay, follow-up medical record review revealed that CRO was isolated from at least one sterile site (blood, abscess, etc) of the patients during hospitalization (Figure 1). Two different CPOs were identified in three patients (P001, P021, P027), one of which was positive for KPC (*K. pneumoniae*) and the other was negative (*Acinetobacter baumannii*) by Xpert Carba-R assay. Noticeably, for one of these three patients (P001), initial colonization screening was positive for IMP instead of KPC, and swab culture was negative for the organism. Similarly, in one patient who developed KPC-producing *K. pneumoniae* infection (P052), the initial colonization screening was positive for NDM rather than KPC.

Overall, a total of 17 patients including 15 male and two female with an average age of 59 years old were found to be colonized or infected with CPO. Sixteen patients had a history of prior hospitalization before admitted to MICU. Invasive operations were performed in 15 out of the 17 patients and five of them had steroid use within 3 months. Thirteen patients had a previous exposure to antibiotics, nine of which had used carbapenems before (Supplementary Table S3). Three of them were infected with CRP with the same carbapenemase as that detected at initial colonization screening by Xpert Carba-R assay, including one whose initial swab culture was negative for the organism, all of which were KPC-producing *K. pneumoniae* (Supplementary Table S2). In six other patients with CR *A*. *baumannii* infection which was negative by Xpert Carba-R assay, OXA-23 or OXA-24 plus OXA-51 carbapenemases were identified in subsequent DNA sequencing (Figure 1). For these patients, initial colonization screening and swab culture revealed diverse results, including three KPC-producing *K. pneumoniae* (P021, P027, P047), two IMP (P001, P003), and one NDM (P098) which was negative in the swab culture. No CRO infection was observed in the remaining two patients (P012, P081) despite a positive colonization result. On the contrary, in two patients whose colonization screening and swab culture were both negative (P038, P039), KPC carbapenemase was identified in the *K. pneumoniae* isolates (Supplementary Table S2). Overall, a significant decreasing trend in the infection rate was shown from 35.7% of baseline to 2.8% in June (*p* = 0.000) (Table 3).

## Discussion

Active surveillance for potential CPO carriers has been recommended as an aid to infection control by CDC to contain the spread of these strains, and has already been adopted as a routine clinical practice in many parts of the world (Albiger et al., 2015). However, limited data is available on the intestinal carriage of CPO in patients in China. So far, only two studies have been published on carbapenemase-producing Enterobacteriaceae (CPE) colonization rates in China, with rates of 2.6% and 0.5% reported in mainland China and Hong Kong, respectively. However, both studies utilized conventional culture-based methods for colonization screening, which was considered time-consuming and laborious, and also had low sensitivity (Kim et al., 2016). To the best of our knowledge, this is the first study to determine the prevalence of CPO intestinal colonization among inpatients in China using Xpert Carba-R assay.

Most studies conducted to date mainly focused on the prevalence of CRE colonization using MacConkey agar to screen for lactose-fermenting colonies (Ben-David et al., 2011; Gijon et al., 2012; Swaminathan et al., 2013; Banach et al., 2014). Subsequent carbapenemase detection was usually based on culture-dependent phenotypic methods, with reported carriage rates varying by geographic locale (Ben-David et al., 2011; Gijon et al., 2012; Swaminathan et al., 2013; Banach et al., 2014). We evaluated the performance of Xpert Carba-R assay using prospectively collected CRE isolates in the first stage of our study. Compared to all four phenotypic methods studied, Xpert Carba-R assay provided more information about specific carbapenemase genes present in each isolate, and accurately identified all the carbapenemase genes tested except for IMP-8 (*n* = 4), which is actually beyond the detection limit of the assay. MHT assay required an overnight incubation and still showed poor sensitivity (87.7%) and specificity (85.2%), as it produced nine false-negative (FN) results for NDM-producing isolates and four false-positive (FP) results, as previously reported (Supplementary Table S1) (Miller et al., 2017). Similarly, the mCIM test also needed an overnight incubation but demonstrated a much better sensitivity (95.9–98.6%) and reported fewer FN results associated with one, two, and three NDM-producing isolates for mCIM-MEM, IPM and ETP, respectively (Supplementary Table S1). For Carba-NP and MHA assays, results can be available within 3 h but both require cumbersome preparation for dedicated reagents, with associated costs and training needs. FN results were observed in five KPC and one IMP isolates for MHA-1h and in one KPC for Carba-NP test (Supplementary Table S1). From the above findings, Xpert Carba-R assay appears to be a good alternative in carbapenemase detection when accuracy and rapidity are taken into consideration. Most importantly, it can provide specific carbapenemase information for each isolate even when multiple resistance genes are present, an outcome also reported in previous studies (Tato et al., 2016), and which cannot be achieved by any of the other phenotypic methods.

In the second stage of our study, we studied the utility of the Xpert Carba-R assay in clinical practice by determining the intestinal CPO colonization rates in patients (by directly testing rectal swabs of hospitalized patients), and coupled this with patient isolation in a MICU ward, in order to contain the spread of CPO. Based on our data, a much higher average CPO colonization rate (11.2%) was observed in our study compared to previous rates of 2.6 and 0.5% reported in mainland China and Hong Kong, respectively (Zhao et al., 2014; Cheng et al., 2016). While geographic locale and time variation may be a possible explanation for the differences in rectal colonization rates, another possible reason is that we calculated the colonization rate based on Xpert Carba-R assay results, whereas the two previous studies utilized conventional culture-based methods for colonization screening, which is considered to be of low sensitivity (Zhao et al., 2014; Cheng et al., 2016; Kim et al., 2016). As our results show, for three IMP, one KPC, and one NDM cases, as detected by Xpert Carba-R assay, no organisms were obtained from swab culture. In another patient whose rectal specimen was positive for NDM by Xpert Carba-R assay, only ESBL was confirmed in subsequent culture of *E. coli*. One possibility is that these patients may have been receiving antimicrobial agents which inhibited the growth of the organisms (Tenover et al., 2013; Tato et al., 2016). Furthermore, it is also possible that the bacterial load in the rectal swab specimens was too low for successful culture on agar plates (Tato et al., 2016). Another possibility is that some or all carbapenemase-producing organisms have fastidious growth requirements, or carry a modified sequence of the target gene that was not expressed or was expressed at low levels (Tenover et al., 2013; Tato et al., 2016).

Despite the high baseline CPO colonization rates, a significant decreasing trend was observed in both the CPO colonization and infection rates during the course of the study period (*p* < 0.05). A total of 15 patients tested positive for CPO colonization initially and 13 of them developed subsequent CRO infection, eight of which had the same carbapenemase (KPC) detected in both the colonizing and infecting bacterial strain, suggesting a pathway from colonization to infection. Two other patients whose initial colonization was with IMP and NDM possessing strains, also developed KPC-producing *K. pneumoniae* infection afterwards. Six patients who were initially CPO colonized (three KPC, two IMP, and one NDM) developed CR *A. baumannii* infection but tested negative for carbapenemase by Xpert Carba-R assay. Subsequent DNA sequencing of these isolates confirmed the presence of OXA-23/24 plus OXA-51 carbapenemases, which are beyond the detection range of Xpert Carba-R assay. Two patients who were negative for CPO colonization also developed CPO infection, with KPC-producing *K. pneumoniae* isolated from the respiratory tract. The route of infection acquisition in these patients was uncertain and requires to be further investigated. Nevertheless, generalizing from the significant decline in CPO colonization and infection rates, it is reasonable to speculate that active surveillance plus patient isolation was an effective measure in the containment of CPO spread.

Our study has several limitations. First, the number of organisms used to establish the sensitivity and specificity of the Xpert Carba-R assay did not cover the entire range of the CP genes that could be detected. No OXA-positive isolate was detected in our study, both in the evaluation and clinical stages due to the scarcity of isolates with this genotype in our setting. At the time of writing, <50 OXA-positive CROs have been reported in previous studies from China (Liu et al., 2015; Guo et al., 2016; Yin et al., 2017). Secondly, we did not use nutrient broth for bacteria enrichment on collected swabs in our study. Consequently, for those swabs that tested positive by Xpert Carba-R assay but negative by direct plate culture, we were unable to trace the root cause. Third, despite the multiple measures recommended by CDC for control of CPE (Centers for Disease Control and Prevention, 2015), we only evaluated the effect of active surveillance plus patient isolation in this tentative study; more preventive strategies should be implemented as soon as possible to better contain the spread of CPE.

In conclusion, our study suggests that the Xpert CARBA-R assay is an easy-to-use and accurate assay for the detection of carbapenemase genes, which allows for rapid identification of patients colonized with CPO directly from rectal swabs. However, the assay is limited in the range of genes detected as it can only detect five carbapenemase genes. Active CPO surveillance using this assay, supplemented with immediate patient isolation, proved to be an effective measure to limit the spread of CPO in a health care setting. The higher CPO colonization rate (11.2%) in the MICU ward of a tertiary care hospital in China compared to previous similar settings (Zhao et al., 2014; Cheng et al., 2016; Kim et al., 2016), is a cause for concern, and calls for immediate action to tackle CPO spread.

## Ethics Statement

The study was approved by the Human Research Ethics Committee of PUMCH and written informed consent was obtained from each patient or their relatives.

## Author Contributions

MZ, QY, and Y-CX conceived and designed the experiments, performed the experiments, analyzed the data, and wrote the paper. YY helped perform the experiments. MZ, TK, BD, JP, XM, YY, GZ, JZ, QY, and Y-CX approved the final version of the manuscript.

### Conflict of Interest Statement

The authors declare that the research was conducted in the absence of any commercial or financial relationships that could be construed as a potential conflict of interest.
