# Supplementary material for: Active Surveillance of Carbapenemase-Producing Organisms (CPO) Colonization With Xpert Carba-R Assay Plus Positive Patient Isolation Proves to Be Effective in CPO Containment
Source: Front Cell Infect Microbiol. 2019 May 14;9:162. doi: 10.3389/fcimb.2019.00162 (PMC6528581; doi:10.3389/fcimb.2019.00162)
Supplement: Supplementary file 1 [file Data_Sheet_1.PDF]

Supplementary Table S1: Detailed results of carbapenemase detection using Xpert Carba-R assay and other phenotypic methods in 100 prospectively collected CRE isolates.

**No.**	**Species**	**Spe**	**Carbapenemase**	**Xpert**	**MHA-1h**	**MHA-2h**	**MHT**	**Carba NP**	**mCIM-MEM**	**mCIM-IMP**	**mCIM-ETP**
D03	*Escherichia coli*	bl	KPC-2	KPC	+	+	+	+	+	+	+
D04	*Klebsiella pneumoniae*	bl	KPC-2	KPC	-	+	+	+	+	+	+
D07	*Klebsiella pneumoniae*	bl	KPC-2	KPC	+	+	+	+	+	+	+
D11	*Citrobacter freundii*	df	IMP-1	IMP1	+	+	+	+	+	+	+
D13	*Escherichia coli*	af	-	ND	-	-	-	-	-	-	-
D16	*Klebsiella pneumoniae*	bl	KPC-2	KPC	+	+	+	+	+	+	+
D18	*Klebsiella pneumoniae*	bl	KPC-2	KPC	+	+	+	+	+	+	+
D21	*Escherichia coli*	bl	KPC-2	KPC	+	+	+	-	+	+	+
D23	*Klebsiella pneumoniae*	af	-	ND	-	-	-	-	-	-	-
D25	*Enterobacter cloacae*	df	NDM-1	NDM	+	+	+	w+	+	+	-
D29	*Escherichia coli*	ur	IMP-1	IMP1	+	+	+	+	+	+	+
D32	*Escherichia coli*	df	-	ND	-	-	-	-	-	-	-
D36	*Enterobacter cloacae*	sp	IMP-1	IMP1	+	+	+	+	+	+	+
D38	*Klebsiella oxytoca*	rs	IMP-1	IMP1	+	+	+	+	+	+	+
D39	*Klebsiella planticola*	bl	-	ND	-	-	-	-	-	-	-
D40	*Enterobacter cloacae*	bl	IMP-4	IMP1	+	+	w +	+	+	+	+

D48	*Enterobacter aerogenes*	sp	-	ND	-	-	-	-	-	-	-
D52	*Klebsiella pneumoniae*	sp	KPC-2	KPC	+	+	+	+	+	+	+
D55	*Enterobacter cloacae*	bi	IMP-8	ND	+	+	+	+	+	+	+
H012	*Serratia marcescens*	ur	-	ND	-	-	-	-	-	-	-
H013	*Klebsiella pneumoniae*	ti	KPC-2	KPC	-	+	+	+	+	+	+
H019	*Escherichia coli*	ur	KPC-2	KPC	+	+	+	+	+	+	+
H024	*Escherichia coli*	ti	-	ND	-	-	-	-	-	-	-
H026	*Escherichia coli*	ti	KPC-2	KPC	+	+	+	+	+	+	+
H027	*Klebsiella pneumoniae*	ab	IMP-26	IMP1	+	+	+	+	+	+	+
H028	*Enterobacter aerogenes*	ab	-	ND	-	-	-	-	-	-	-
H029	*Klebsiella pneumoniae*	ur	-	ND	-	-	-	-	-	-	-
H031	*Klebsiella pneumoniae*	ur	IMP-8	ND	+	+	+	+	+	+	+
H034	*Klebsiella planticola*	ur	IMP-4	IMP1	+	+	+	+	+	+	+
H035	*Escherichia coli*	ur	KPC-2	KPC	+	+	+	+	+	+	+
H044	*Klebsiella pneumoniae*	ur	-	ND	-	-	-	-	-	-	-
H054	*Klebsiella oxytoca*	ab	IMP-4	IMP1	+	+	+	+	+	+	+
H057	*Klebsiella pneumoniae*	ti	KPC-2	KPC	+	+	+	+	+	+	+
H067	*Escherichia coli*	ti	-	ND	-	-	-	-	-	-	-
H075	*Escherichia coli*	ab	KPC-2	KPC	+	+	+	+	+	+	+
H090	*Serratia marcescens*	ab	KPC-2	KPC	-	+	+	+	+	+	+

H102	*Klebsiella pneumoniae*	ur	KPC-2	KPC	+	+	+	+	+	+	+
H103	*Klebsiella pneumoniae*	ur	KPC-2	KPC	+	+	+	+	+	+	+
H106	*Escherichia coli*	ur	KPC-2	KPC	+	+	+	+	+	+	+
L001	*Klebsiella pneumoniae*	sp	NDM-1	NDM	+	+	-	+	+	+	+
L002	*Enterobacter cloacae*	ws	NDM-1	NDM	+	+	+	+	+	+	+
L003	*Klebsiella pneumoniae*	sp	NDM-1	NDM	+	+	-	+	+	+	+
L004	*Klebsiella pneumoniae*	sp	NDM-1	NDM	+	+	-	+	+	+	+
L005	*Enterobacter cloacae*	ca	NDM-1	NDM	+	+	w +	+	+	+	+
L006	*Enterobacter cloacae*	sp	NDM-1	NDM	+	+	w +	+	+	+	+
L007	*Enterobacter cloacae*	sp	IMP-26	IMP1	+	+	+	+	+	+	+
L008	*Enterobacter cloacae*	bl	NDM-1	NDM	+	+	+	+	+	+	+
L012	*Klebsiella oxytoca*	bl	NDM-1	NDM	+	+	-	+	+	+	+
L013	*Klebsiella pneumoniae*	bi	NDM-1	NDM	+	+	-	+	+	+	+
L014	*Klebsiella pneumoniae*	sp	NDM-1	NDM	+	+	-	+	+	+	+
L018	*Enterobacter cloacae*	bl	-	ND	-	-	-	-	-	-	-
L019	*Enterobacter cloacae*	sf	-	ND	-	-	-	-	-	-	-
L029	*Enterobacter aerogenes*	ab	KPC-2	KPC	+	+	+	+	+	+	+
L030	*Klebsiella oxytoca*	bl	IMP-4	IMP1	+	+	w +	+	+	+	+
L031	*Klebsiella oxytoca*	ur	-	ND	-	-	-	-	-	-	-
L033	*Klebsiella pneumoniae*	bl	IMP-4	IMP1	+	+	w +	+	+	+	+

L040	*Klebsiella pneumoniae*	sp	KPC-2	KPC	-	+	+	+	+	+	+
L044	*Klebsiella pneumoniae*	ur	-	ND	-	-	-	-	-	-	-
L048	*Klebsiella pneumoniae*	sp	KPC-2	KPC	+	+	+	+	+	+	+
L052	*Klebsiella pneumoniae*	ur	KPC-2	KPC	+	+	+	+	+	+	+
L055	*Escherichia coli*	bl	-	ND	-	-	-	-	-	-	-
L060	*Klebsiella pneumoniae*	sp	KPC-2	KPC	+	+	+	+	+	+	+
L068	*Proteus mirabilis*	ur	KPC-2	KPC	+	+	+	+	+	+	+
L070	*Klebsiella pneumoniae*	bl	KPC-2	KPC	+	+	w +	+	+	+	+
L085	*Enterobacter cloacae*	bl	NDM-1	NDM	+	+	+	+	+	+	+
L086	*Enterobacter cloacae*	BALF	NDM-1	NDM	+	+	w +	+	+	-	-
L087	*Enterobacter cloacae*	bl	NDM-1	NDM	+	+	+	+	-	-	-
L090	*Klebsiella pneumoniae*	ur	KPC-2	KPC	+	+	+	+	+	+	+
L094	*Klebsiella pneumoniae*	ws	IMP-8	ND	-	+	w +	+	+	+	+
L095	*Enterobacter cloacae*	ur	KPC-2	KPC	+	+	+	+	+	+	+
L096	*Citrobacter freundii*	CSF	NDM-1	NDM	+	+	w +	+	+	+	+
L098	*Escherichia coli*	sf	-	ND	-	-	-	-	-	-	-
L100	*Enterobacter cloacae*	ur	NDM-1	NDM	+	+	+	+	+	+	+
L104	*Escherichia coli*	bl	NDM-1	NDM	+	+	-	+	+	+	+
L105	*Enterobacter cloacae*	ws	IMP-1, VIM-1	IMP, VIM	+	+	+	+	+	+	+
L106	*Enterobacter cloacae*	sp	IMP-1, VIM-1	IMP, VIM	+	+	+	+	+	+	+

L107	*Klebsiella pneumoniae*	sp	IMP-4	IMP1	+	+	+	+	+	+	+
L109	*Klebsiella oxytoca*	bl	KPC-2	KPC	+	+	+	+	+	+	+
L110	*Escherichia coli*	sp	NDM-1	NDM	+	+	+	+	+	+	+
L112	*Klebsiella pneumoniae*	sp	IMP-4	IMP1	+	+	w +	+	+	+	+
L121	*Enterobacter cloacae*	ca	KPC-2, IMP-4	KPC, IMP	+	+	+	+	+	+	+
L127	*Providencia rette*	gf	-	ND	-	-	-	-	-	-	-
L140	*Enterobacter cloacae*	ur	-	ND	-	-	-	-	-	-	-
L143	*Klebsiella pneumoniae*	bl	KPC-2	KPC	+	+	w +	+	+	+	+
L150	*Klebsiella oxytoca*	df	KPC-2, IMP-4	KPC, IMP	+	+	+	+	+	+	+
L151	*Escherichia coli*	df	NDM-1	NDM	+	+	-	+	+	+	+
L153	*Escherichia coli*	bl	NDM-1	NDM	+	+	-	+	+	+	+
R02	*Enterobacter cloacae*	af	-	ND	-	-	-	-	-	-	-
R17	*Enterobacter cloacae*	af	-	ND	-	-	-	-	-	-	-
R35	*Klebsiella pneumoniae*	af	-	ND	-	-	w +	-	-	-	-
R37	*Klebsiella pneumoniae*	af	KPC-2	KPC	+	+	+	+	+	+	+
Y047	*Klebsiella pneumoniae*	bl	IMP-8	ND	+	+	+	+	+	+	+
Y060	*Escherichia coli*	sp	-	ND	-	-	w +	-	-	+	-
Y062	*Citrobacter freundii*	ur	IMP-4	IMP1	+	+	+	+	+	+	+
Y067	*Klebsiella pneumoniae*	sp	-	ND	-	-	w +	-	-	-	-
Y068	*Klebsiella pneumoniae*	sp	-	ND	-	-	-	-	-	-	-

Y070	*Klebsiella pneumoniae*	sp	KPC-2	KPC	-	+	+	+	+	+	+
Y074	*Escherichia coli*	sp	-	ND	-	-	-	-	-	-	-
Y076	*Klebsiella pneumoniae*	bl	IMP-4	IMP1	+	+	+	+	+	+	+
Y078	*Citrobacter freundii*	bl	-	ND	-	-	w +	-	-	-	-

Spe: specimen; ab: abdominal fluid; af: ascitic fluid; BALF: bronchoalveolar lavage fluid; bi: bile; bl: blood; ca: catheter; CSF: cerebrospinal fluid; df: drainage fluid; gf: gastric fluid; rs: rectal swab; sf: shunt fluid; sp: sputum; ti: tissue; ur: midstream urine; ws: wound swab.

w+: Results that were interepreted as weak positive and were classified as positive for analysis.

Supplementary Table S2: Detailed information of Xpert Carba-R assay results with prospective rectal swab specimens.

Patient No.	Adm	Dis	Colonization Screening						Follow-up Infection		
			Xpert	ST	Swab culture	IPM	MEM	ETP	Strain isolated	Xpert	Carbapenemase Seq
P001	12-Jan	8-Feb	IMP1	4	ND				*Klebsiella pneumoniae*	KPC	KPC
									Acinetobacter baumannii	ND	OXA-23, OXA-51
P002	1-Jan	30-Jun	ND	26	ND						
P003	8-Apr	24-Apr	IMP1	3	ND				*Acinetobacter baumannii*	ND	OXA-23, OXA-51
P004	1-Jan	3-Mar	ND	9	*Candida glabrata*						
P005	1-Jan	27-Feb	ND	9	ND						
P006	11-Feb	5-Apr	ND	8	*Candida tropicalis*						
P007	28-Apr	16-Jun	ND	8	*Escherichia coli*	S	S	S			
P008	16-Apr	26-May	ND	6	*Escherichia coli*	S	S	S			
P009	22-May	4-Jul	ND	6	*Klebsiella pneumoniae*	S	S	S			
P010	24-May	3-Jul	ND	6	ND						
P011	18-May	24-Jun	ND	6	*Escherichia coli*	S	S	S			
P012	25-	7-Jun	IMP1	2	ND						

	May										
P013	3-Jan	31-Jan	ND	5	*Escherichia coli*	S	S	S			
P014	10-Apr	8-May	ND	5	*Proteus mirabilis*	S	S	S			
P015	1-Jan	3-Feb	ND	5	ND						
P016	23-Apr	22-May	ND	5	*Escherichia coli*	S	S	S			
P017	21-Mar	22-May	KPC	9	*Klebsiella pneumoniae*	R	R	R	*Klebsiella pneumoniae*	KPC	KPC
P018	1-Jan	24-Jan	ND	4	ND						
P019	17-Mar	7-Apr	ND	4	*Pseudomonas aeruginosa*	S	S	R			
P020	1-Jan	3-Feb	KPC	5	*Klebsiella pneumoniae*	R	R	R	*Klebsiella pneumoniae*	KPC	KPC
P021	1-Jan	25-Jan	KPC	4	*Klebsiella pneumoniae*	R	R	R	*Klebsiella pneumoniae*	KPC	KPC
									Acinetobacter baumannii	ND	OXA-23, OXA-51
P022	16-Mar	8-Apr	ND	4	*Escherichia coli*	S	S	S			
P023	9-Apr	5-May	ND	4	*Escherichia coli*	S	S	S			
P024	27-May	20-Jun	ND	4	*Escherichia coli*	S	S	S			
P025	1-Jan	23-Jan	ND	4	ND						
P026	14-Mar	9-Apr	ND	4	*Escherichia coli*	S	S	S			
P027	1-Mar	26-Mar	KPC	4	*Klebsiella pneumoniae*	R	R	R	*Klebsiella pneumoniae*	KPC	KPC
									Acinetobacter	ND	OXA-23, OXA-51

									baumannii		
P028	27-Jan	19-Feb	ND	4	*Escherichia coli*	S	S	S			
P029	25-Jan	21-Feb	ND	4	*Escherichia coli*	S	S	S			
P030	25-Apr	22-May	ND	4	*Escherichia coli*	S	S	S			
P031	16-Jun	21-Jul	ND	3	*Klebsiella pneumoniae*	S	S	S			
P032	7-Jun	26-Jun	ND	3	*Escherichia coli*	S	S	S			
P033	10-Jun	4-Jul	ND	3	ND						
P034	2-Apr	20-Apr	ND	3	*Klebsiella pneumoniae*	S	S	S			
P035	6-Feb	23-Feb	ND	3	ND						
P036	5-Apr	30-Apr	KPC	4	*Klebsiella pneumoniae*	R	R	R	*Klebsiella pneumoniae*	KPC	KPC
P037	2-Jun	16-Jun	ND	3	*Escherichia coli*	S	S	S			
P038	1-Jan	20-Jan	ND	3	ND				*Klebsiella pneumoniae*	KPC	KPC
P039	18-Feb	6-Mar	ND	3	ND				*Klebsiella pneumoniae*	KPC	KPC
P040	18-Mar	6-Apr	ND	3	ND						
P041	27-Mar	11-Apr	KPC	3	*Klebsiella pneumoniae*	R	R	R	*Klebsiella pneumoniae*	KPC	KPC
P042	1-Jan	16-Jan	KPC	3	*Klebsiella pneumoniae*	R	R	R	*Klebsiella pneumoniae*	KPC	KPC
P043	1-Jan	16-Jan	KPC	3	ND				*Klebsiella pneumoniae*	KPC	KPC
P044	12-	5-Jul	ND	3	*Klebsiella pneumoniae*	S	S	S			

	Jun										
P045	5-May	25-May	ND	3	*Escherichia coli*	S	S	S			
P046	16-May	5-Jun	ND	3	*Enterococcus* spp.						
P047	6-Apr	13-Apr	KPC	2	*Klebsiella pneumoniae*	R	R	R	*Acinetobacter baumannii*	ND	OXA-24, OXA-51
P048	29-Mar	5-Apr	ND	2	*Escherichia coli*	S	S	S			
P049	31-May	8-Jun	ND	2	*Escherichia coli*	S	S	S			
P050	26-Apr	3-May	ND	2	*Escherichia coli*	S	S	S			
P051	26-Jan	6-Feb	ND	2	*Klebsiella pneumoniae*	S	S	S			
P052	16-Jun	15-Aug	NDM	3	*Klebsiella pneumoniae*	R	R	R	*Klebsiella pneumoniae*	KPC	NDM
P053	24-Apr	2-May	ND	2	*Escherichia coli*	S	S	S			
P054	22-Mar	29-Mar	ND	2	*Escherichia coli*	S	S	S			
P055	18-May	31-May	ND	2	*Klebsiella pneumoniae*	S	S	S			
P056	9-Apr	20-Apr	ND	2	*Escherichia coli*	S	S	S			
P057	23-Apr	4-May	ND	2	*Enterococcus faecium*						

P058	17-Jun	26-Jun	ND	2	*Escherichia coli*	S	S	S
P059	1-Jan	11-Jan	ND	2	ND			
P060	1-Jan	13-Jan	ND	2	ND			
P061	8-May	16-May	ND	2	*Klebsiella pneumoniae*	S	S	S
P062	23-May	31-May	ND	2	*Escherichia coli*	S	S	S
P063	8-Jun	20-Jun	ND	2	*Escherichia coli*	S	S	S
P064	23-May	2-Jun	ND	2	*Escherichia coli*	S	S	S
P065	14-May	21-May	ND	2	*Escherichia coli*	S	S	S
P066	1-Jan	13-Jan	ND	2	*Klebsiella pneumoniae*	S	S	S
P067	23-Mar	31-Mar	ND	2	*Escherichia coli*	S	S	S
P068	14-May	22-May	ND	2	*Escherichia coli*	S	S	S
P069	9-Apr	18-Apr	ND	2	*Escherichia coli*	S	S	S
P070	23-Jun	12-Jul	ND	2	ND			
P071	30-Mar	8-Apr	ND	2	*Klebsiella pneumoniae*	S	S	S
P072	1-Jan	12-Jan	ND	2	ND			
P073	22-Feb	2-Mar	ND	2	*Escherichia coli*	S	S	S

P074	23-Jun	2-Jul	ND	2	*Proteus mirabilis*	S	S	S
P075	28-May	8-Jun	ND	2	*Klebsiella pneumoniae*	S	S	S
P076	21-Jun	19-Jul	ND	2	*Escherichia coli*	S	S	S
P077	24-Mar	31-Mar	ND	2	ND			
P078	19-Mar	1-Apr	ND	2	ND			
P079	19-Jun	17-Jul	ND	2	*Escherichia coli*	S	S	S
P080	8-Apr	19-Apr	ND	2	*Escherichia coli*	S	S	S
P081	18-Jun	21-Jun	NDM	1	*Escherichia coli*	S	S	S
P082	19-Apr	26-Apr	ND	2	ND			
P083	26-Jan	3-Feb	ND	2	*Escherichia coli*	S	S	S
P084	2-Mar	13-Mar	ND	2	ND			
P085	12-Mar	14-Mar	ND	1	*Klebsiella pneumoniae*	S	S	S
P086	18-Feb	21-Feb	ND	1	*Escherichia coli*	S	S	S
P087	20-May	25-May	ND	1	*Enterococcus* spp.			

P088	27-Jun	30-Jun	ND	1	*Escherichia coli*	S	S	S			
P089	21-Mar	24-Mar	ND	1	*Escherichia coli*	S	S	S			
P090	16-Apr	19-Apr	ND	1	*Escherichia coli*	S	S	S			
P091	30-Apr	5-May	ND	1	ND						
P092	3-May	5-May	ND	1	*Klebsiella pneumoniae*	S	S	S			
P093	16-Jun	18-Jun	ND	1	ND						
P094	9-Mar	15-Mar	ND	1	*Escherichia coli*	S	S	S			
P095	25-Jan	31-Jan	ND	1	ND						
P096	6-Apr	9-Apr	ND	1	*Escherichia coli*	S	S	S			
P097	8-Apr	9-Apr	ND	1	*Candida albicans*						
P098	21-May	23-May	NDM	1	ND				*Acinetobacter baumannii*	ND	OXA-23, OXA-51
P099	13-Apr	17-Apr	ND	1	ND						
P100	12-Jan	14-Jan	ND	1	*Klebsiella pneumoniae*	S	S	S			
P101	20-Apr	24-Apr	ND	1	*Escherichia coli*	S	S	S			
P102	17-	23-	ND	1	*Klebsiella pneumoniae*	S	S	S			

	May	May						
P103	11-Feb	16-Feb	ND	1	*Escherichia coli*	S	S	S
P104	20-May	21-May	ND	1	*Candida* spp.			
P105	1-May	6-May	ND	1	*Escherichia coli*	S	S	S
P106	7-May	10-May	ND	1	*Enterobacter cloacae*	S	S	S
P107	26-Jun	29-Jun	ND	1	*Escherichia coli*	S	S	S
P108	22-Feb	27-Feb	ND	1	ND			
P109	26-May	26-May	ND	1	*Escherichia coli*	S	S	S
P110	19-Apr	21-Apr	ND	1	*Escherichia coli*	S	S	S
P111	20-Jun	21-Jun	ND	1	ND			
P112	17-Feb	21-Feb	ND	1	*Escherichia coli*			
P113	24-Mar	27-Mar	ND	1	ND			
P114	16-Feb	21-Feb	ND	1	*Escherichia coli*	S	S	S
P115	17-	20-Apr	ND	1	*Escherichia coli*	S	S	S

	Apr							
P116	1-Jun	2-Jun	ND	1	*Escherichia coli*	S	S	S
P117	11-Feb	14-Feb	ND	1	*Enterococcus faecium*			
P118	6-May	11-May	ND	1	*Escherichia coli*	S	S	S
P119	4-May	9-May	ND	1	*Klebsiella pneumoniae*	S	S	S
P120	21-Apr	25-Apr	ND	1	*Enterococcus faecium*			
P121	8-Jun	9-Jun	ND	1	*Escherichia coli*	S	S	S
P122	26-Apr	29-Apr	ND	1	*Escherichia coli*	S	S	S
P123	19-Jun	20-Jun	ND	1	*Klebsiella pneumoniae*	S	S	S
P125	10-Apr	11-Apr	ND	1	*Escherichia coli*	S	S	S
P126	1-Apr	5-Apr	ND	1	*Enterococcus faecalis*			
P127	8-Jun	13-Jun	ND	1	*Escherichia coli*	S	S	S
P128	5-Apr	10-Apr	ND	1	ND			
P129	11-Apr	12-Apr	ND	1	*Escherichia coli*	S	S	S
P130	21-May	25-May	ND	1	ND			
P131	17-Jun	18-Jun	ND	1	*Escherichia coli*	S	S	S

P132	9-Apr	15-Apr	ND	1	*Escherichia coli*	S	S	S
P133	11-Apr	17-Apr	ND	1	*Acinetobacter baumannii*	R	R	R
P134	15-Jun	16-Jun	ND	1	*Enterococcus faecium*			
P135	27-Jun	3-Jul	ND	1	*Escherichia coli*	S	S	S

Adm: admission date; Dis: discharge date; ST: screening times; ND: not detected.

Supplementary Table S3: Detailed clinical information of the 17 patients colonized and/or infected with CRO.

**Patient No.**	**Sex**	**Age**	**PH**	**CN**	**IF**	**Underlying diseases**	**Invasive operations**				**Steroid use within three months**	**Antibiotic use within three months**				
							**CVC**	**MV**	**T**	**UC**		**Carbapenems**	**Aminoglycosides**	**Glycopeptides**	**Quinolones**	**Penicillins**
P001	M	55	Y	Pos	Pos	DB, HL	Y	Y	Y	Y	Y	Y		Y	Y	Y
P003	M	30	N	Pos	Pos	None	N	Y	Y	Y						
P012	M	68	Y	Pos	Neg	DB, HT, RF	N	Y	Y	N	Y			Y		
P017	M	72	Y	Pos	Pos	EC	N	N	Y	N						
P020	M	40	Y	Pos	Pos	HT	Y	Y	Y	N		Y	Y	Y	Y	
P021	M	69	Y	Pos	Pos	HT	N	N	N	N		Y				
P027	M	54	Y	Pos	Pos	HT	Y	Y	N	N					Y	Y
P036	F	31	Y	Pos	Pos	SLE	Y	N	N	N						
P038	M	65	Y	Neg	Pos	RF	Y	Y	Y	N	Y				Y	Y
P039	M	83	Y	Neg	Pos	HT, HF, RF	Y	Y	Y	N		Y			Y	
P041	M	71	Y	Pos	Pos	HT, HP, A	N	Y	Y	N		Y				
P042	M	61	Y	Pos	Pos	DB, HT	N	Y	Y	N		Y		Y		Y
P043	F	67	Y	Pos	Pos	HP, HF, RF	Y	N	Y	N						
P047	M	53	Y	Pos	Pos	HT, HF, RF	Y	Y	Y	N		Y			Y	
P052	M	90	Y	Pos	Pos	HF, RF	Y	Y	Y	N	Y	Y		Y		
P081	M	45	Y	Pos	Neg	HHC, HL	N	N	N	N					Y	
P098	M	63	Y	Pos	Pos	RF	N	Y	Y	N	Y	Y				

PH: prior hospitalization before admission to MICU, CN: colonization, IF: infection, DB: diabetes, HL: hyperlipemia HT: hypertension, RF: renal failure, EC: esophageal carcinoma, SLE: systemic lupus erythematosus, HF: heart failure, HP: hypoproteinemia, A: anemia, HHC: hyperhomocysteinemia, CVC: central venous catheter use, MV: mechanical ventilation, T: tracheostomy, UC: urinary catherization, Y: yes.
